# Supplementary material for: Inactivation of Bacteriophage ɸ6 and SARS-CoV-2 in Antimicrobial Surface Tests
Source: Viruses. 2023 Aug 29;15(9):1833. doi: 10.3390/v15091833 (PMC10535131; doi:10.3390/v15091833)
Supplement: Supplementary file 1 [file viruses-15-01833-s001.zip › viruses-2555754-supplementary.pdf]

Results for ϕ6

Table S1. Log-reduction of the infectious viral particles of bacteriophage ϕ6 after the different temporal incubations on the tested specimens and test validity. The logarithmic decrease was calculated for all time points by the applied load on each specimen. For the results of the test validity, all values (n=6) of the two independent runs were considered.

	0 h	10 min	1 h	24 h	Test validity
**Reference**	0.15 $\log_{10}$	0.15 $\log_{10}$	0.54 $\log_{10}$	5.28 $\log_{10}$	0.08
**Cu2**	0.72 $\log_{10}$	5.51 $\log_{10}$	5.51 $\log_{10}$	5.51 $\log_{10}$	0.15
**Cu1**	0.38 $\log_{10}$	5.13 $\log_{10}$	5.34 $\log_{10}$	5.34 $\log_{10}$	0.14
**Cu0.5**	0.17 $\log_{10}$	4.22 $\log_{10}$	5.34 $\log_{10}$	5.34 $\log_{10}$	0.10
**Cu0.25**	0.18 $\log_{10}$	2.61 $\log_{10}$	5.43 $\log_{10}$	5.43 $\log_{10}$	0.14

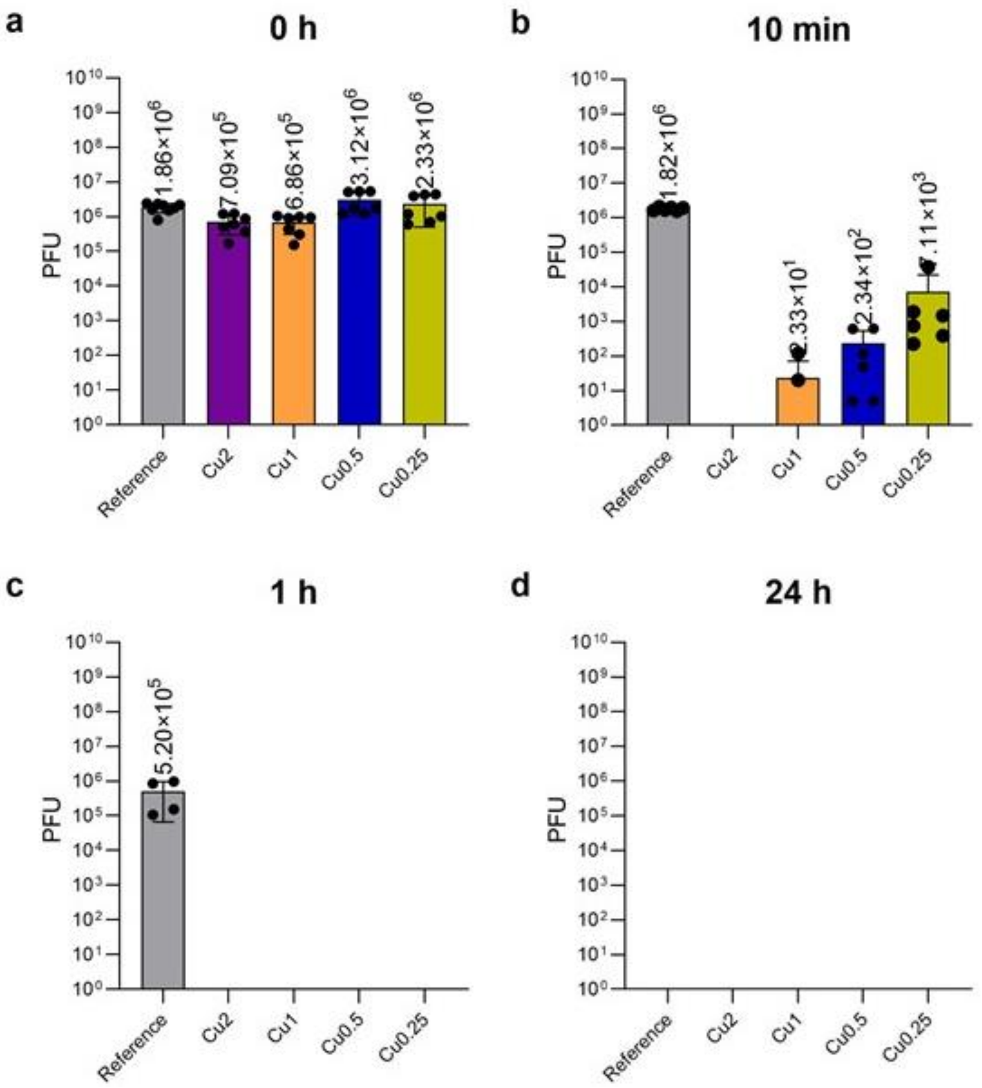

Figure S1. Data from plaque assay with bacteriophage ϕ6 presented by the incubation periods: 0 h (a), 10 min (b), 1 h (c) and 24 h (d). All results consist of triplicates from two independent runs (n=6). The only exception is the reference sample at 1 h of incubation, since two samples were not evaluable (n=4). The limit of detection (10 PFU) were not included in the diagrams above. Therefore, results without a shown bar are attributed to plates were no plaques were countable.

Results for SARS-CoV-2

Table S2. Log-reduction of SARS-CoV-2 RNA (virus input) after the different temporal incubations on the tested specimenss. The logarithmic decrease was calculated for all time points based on the positive control (2.52 x 106 virus copies) performed.

	**0 h**	**1 h**	**24 h**
**Reference**	1.1 $\log_{10}$	2.2 $\log_{10}$	2.9 $\log_{10}$
**Cu2**	1.1 $\log_{10}$	5.1 $\log_{10}$	> 7 $\log_{10}$
**Cu1**	1.1 $\log_{10}$	5.5 $\log_{10}$	> 7 $\log_{10}$
**Cu0.5**	1.1 $\log_{10}$	2.3 $\log_{10}$	> 7 $\log_{10}$
**Cu0.25**	1.1 $\log_{10}$	2.1 $\log_{10}$	> 7 $\log_{10}$

Table S3. Log-reduction of the infectious viral particles of SARS-CoV-2 (at t=48 post infection) after the different incubation periods on the tested specimens. The logarithmic decrease was calculated for all time points based on the positive control performed.

	**0 h**	**1 h**	**24 h**
**Reference**	1.1 $\log_{10}$	< 1 $\log_{10}$	6.1 $\log_{10}$
**Cu2**	< 1 $\log_{10}$	> 7 $\log_{10}$	6.7 $\log_{10}$
**Cu1**	< 1 $\log_{10}$	6.3 $\log_{10}$	> 7 $\log_{10}$
**Cu0.5**	< 1 $\log_{10}$	6.1 $\log_{10}$	> 7 $\log_{10}$
**Cu0.25**	< 1 $\log_{10}$	1.1 $\log_{10}$	> 7 $\log_{10}$

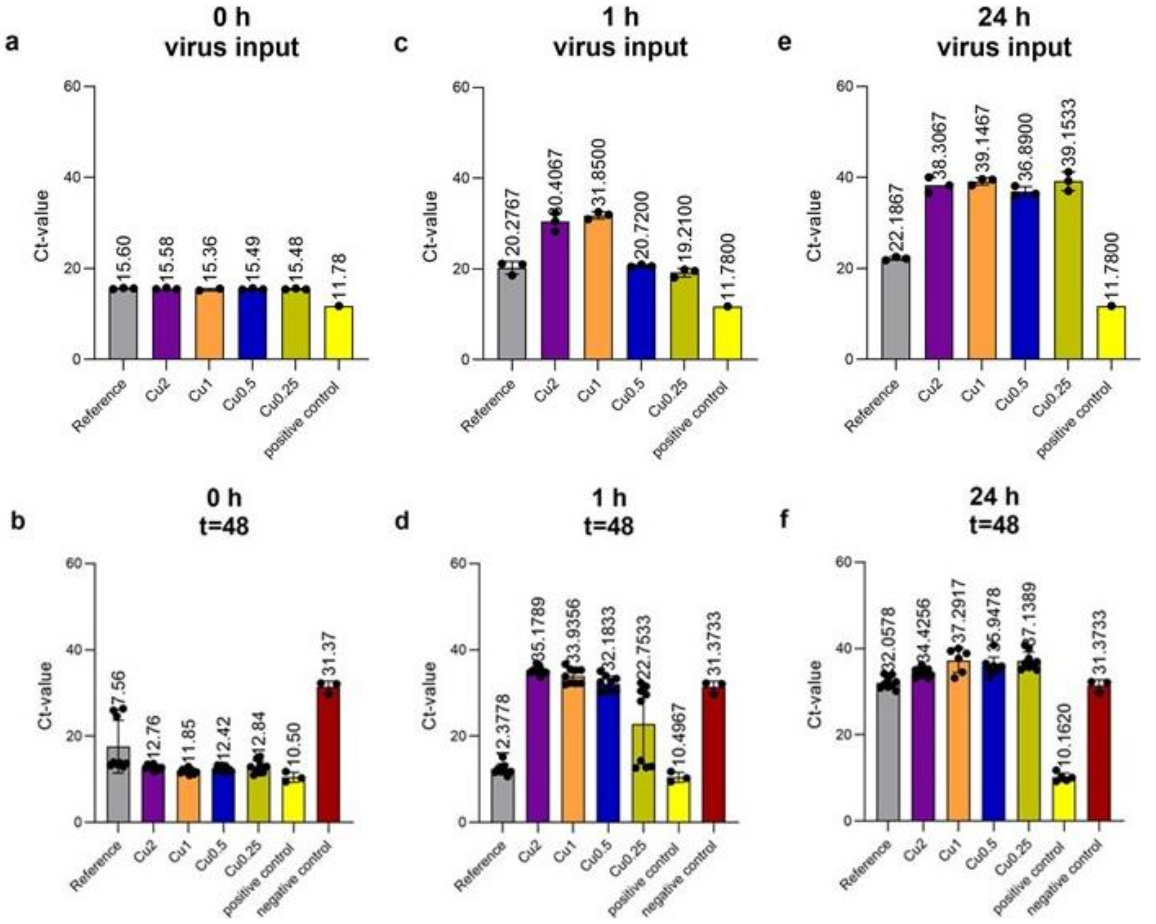

Figure S2. Data from RT-qPCR performed for SARS-CoV-2 separated by the incubation periods: 0 h (a,b), 1 h (c,d) and 24 h (e,f). CT-values are shown for virus input (amount of virus RNA used for the infection, n=3, a,c,e) and at 48 hours after infection (t=48) of VeroE6 cells (virus neutralization test, n=9, b,d,f). Positive controls reflect amount of SARS-CoV-2 RNA loaded onto coated surfaces and negative controls medium without virus added.
